# Trachoma and the Importance of Sexual Infective Route in Developed Countries. Comment on Gallenga et al. Why the SAFE—*S* Strategy for Trachoma? Are *Musca sorbens* or *Scatophaga stercoraria* Really the Culprit?—A Brief Historical Review from an Italian Point of View. *Pathogens* 2023, *12*, 1419

**DOI:** 10.3390/pathogens13050413

**Published:** 2024-05-16

**Authors:** Damian Garcia-Teillard, Salvador García-Delpech, Patricia Udaondo

**Affiliations:** 1 Hospital Universitario Punta de Europa, 11207 Algeciras, Spain; 2 Clínica Aiken, 46004 Valencia, Spain

We have read the review article entitled “Why the SAFE—S Strategy for Trachoma? Are Musca sorbens or Scatophaga stercoraria Really the Culprit?—A Brief Historical Review from an Italian Point of View.” [1], We want to point out an aspect that may have been forgotten regarding the investigation of Trachoma vectors, which could be a key factor when it comes to understanding Gallenga’s conclusion regarding Trachoma and *Chlamydia trachomatis*’s epidemiology in higher-income countries and the need for sexual behavior control.

The incidence of visual impairment due to Trachoma is lower in European and Latin American countries in comparison to African countries [2,3], due to Trachoma’s association with rural and lower-income areas [1], as has been clearly pointed out in the discussed review article. As stated by the authors, Trachoma’s global elimination has been proposed for 2030 [3]. The appearance of “fly killers” has induced a decrease in the total population of eye-seeking flies, not only in highest-income countries but also in rural and lower-income areas [4]. 

Ailie Robinson, Julie Bristow et al. pointed out in their revision “*Responses of the putative trachoma vector*, *Musca sorbens*, *to volatile semiochemicals from human faeces*” [4] that flies are more attracted to human feces rather than other animal feces, due to their characteristic odor. They suggest that, by properly isolating and segregating these odors, we could attract these flies to “traps” to be eliminated. This could be a potential alternative to chemical fly killers that have been key in reducing the population of eye-seeking flies, as well as another alternative for fly population control. 

Despite the previously shared information, when we think of higher-income countries’ levels of sanitization, it is difficult to imagine eye-seeking flies being attracted to human feces and therefore being the main vector for the infection. Yet, ocular infections caused by *Chlamydia trachomatis* (*Ct*), apart from Trachoma, are still persistent in these countries [1]. Indeed, as Gallenga et al. remark, *Ct* has not been fully eradicated, probably due to the sexual infective route [1]. *Ct* serovars D and K cause genital disease [5,6] and have tropism for ocular epithelial cells [5]. This could be key in understanding why ocular *Ct* infections have not been fully eradicated [4] in high-income countries, despite high levels of sanitization.

In fact, the WHO (World Health Organization) has reported that over one million people contract sexually transmitted infections daily [7]. Thus, *Ct* is far from being eliminated unless a sexual strategy is implemented in the SAFE program. As a result, we can only agree with Gallenga’s conclusion in this review article.
